# Dislodged cranial bone due to application of a MAYFIELD™ skull clamp in a patient with a previous history of craniotomy

**DOI:** 10.1186/s40981-020-00319-6

**Published:** 2020-02-12

**Authors:** Taiji Okamoto, Yoshimune Osaka, Yoshihisa Morita

**Affiliations:** Department of Anesthesiology, Kawasaki Municipal Hospital, 12-1 Shinkawa Street, Kawasaki-ku, Kawasaki City, Kanagawa 210-0013 Japan

**Keywords:** Dislodged skull, MAYFIELD™ skull clamp, Spinal surgery

To the Editor,

The MAYFIELD™ three-pin skull clamp (MAYFIELD™, OHWA TSUSHO CO., LTD., Tokyo, Japan) allows excellent cranial stabilization during head and neck surgery. However, potentially serious and/or life-threatening complications, such as scalp lacerations and depressed skull fractures, can rarely occur [1–3]. We encountered dislodgement of a cranial bone caused by a skull clamp in a patient with a previous history of craniotomy.

A 53-year-old male (height 163 cm, weight 90 kg, American Society of Anesthesiologists Physical Status II) was scheduled to undergo posterior cervical spinal fusion. He had undergone craniotomy 20 years earlier, but no precise details could be obtained. After induction of general anesthesia and tracheal intubation, a skull clamp was applied on the safe zones of the temporal region [3] with an indicator to notify the screwing power to the operator (appropriate pressure could be maintained with the standard 60-lb torque screw), and the patient was placed in the prone position on a Jackson spinal table (MIZUHO Co., Ltd., Tokyo, Japan). The surgery lasted for 328 min and the patient’s hemodynamic/respiratory status was stable. Skull deformity in the left temporal region was noted after removal of the drapes following surgery. Skull X-ray revealed that bilateral parietal regions had been replaced with artificial bone, which was loosely fixed to the left temporal region of the patient’s skull, and the artificial bone was dislodged by the skull clamp (Fig. 1). Resultantly, open reduction and fixation of the skull were performed. The total anesthesia time was 687 min. The patient regained consciousness at the completion of surgery and was extubated the following day. His subsequent clinical course was uneventful.

**Fig. 1:**
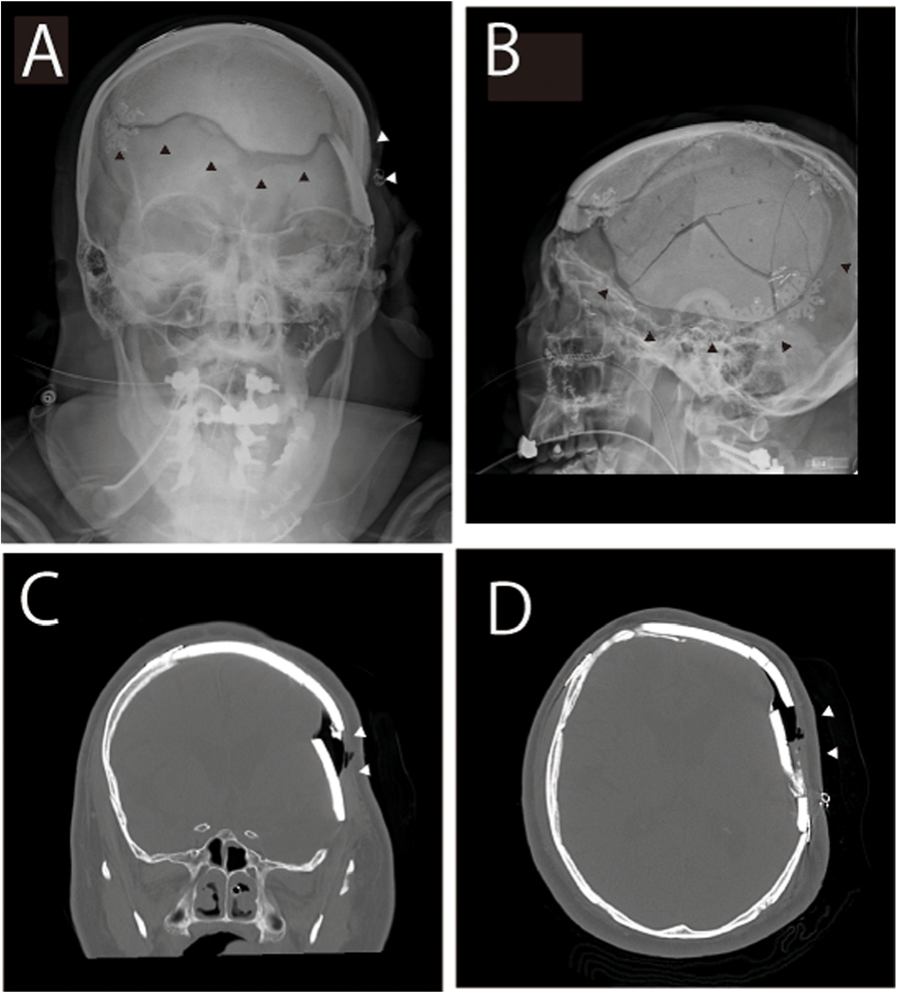
Skull X-ray and CT images. **a** Coronal view and **b** sagittal view X-ray image showing dislodgement of the left temporal region of the skull (white arrows) and the artificial cranial bones (black arrows) loosely fixed to the patient’s skull. **c** Coronal view and **d** transverse view CT image showing dislodgement of the skull (white allows)

Depressed skull fracture is one of the most serious complications that can arise from the application of skull clamps that could be prevented by placing the skull pins outside the areas of thin cranial bone (temporal squama, frontal sinus, and coronal suture) [1]. Although CT is not routinely performed in adults before spinal surgery, in our patient, the skull had been partially replaced with an artificial bone that had not been sufficiently firmly fixed (Fig. 1), and compression by the skull clamp led to skull dislodgement in this patient. Fortunately, there was no brain damage. For appropriate head pin fixation in a patient with a history of cranial surgery, the condition of the skull should be precisely evaluated prior to any future surgery, e.g., by a preoperative head CT. In addition, we should also have observed the intraoperative skull position more carefully during the surgery or should have used the horseshoe-type head rest instead, even though head fixation with the latter is inferior to that with a skull clamp.

Our case highlights the need to precisely evaluate the condition of the skull before installing a skull clamp in patients with a previous history of craniotomy.

## Data Availability

Not applicable
